# A Case of Atypical Bartonellosis in a 4-Year-Old Immunocompetent Child

**DOI:** 10.3390/microorganisms9050950

**Published:** 2021-04-28

**Authors:** Chiara Sodini, Elena Mariotti Zani, Francesco Pecora, Cristiano Conte, Viviana Dora Patianna, Giovanni Prezioso, Nicola Principi, Susanna Esposito

**Affiliations:** 1 Pediatric Clinic, Pietro Barilla Children’s Hospital, University of Parma, Via Gramsci 14, 43126 Parma, Italy; chiara.sodini@gmail.com (C.S.); elena.mariottizani@unipr.it (E.M.Z.); cescopec@hotmail.it (F.P.); cristianoconte1993@gmail.com (C.C.); viviana.patianna@gmail.com (V.D.P.); gprezioso@ao.pr.it (G.P.); 2 Università degli Studi di Milano, 20122 Milan, Italy; nicola.principi@unimi.it

**Keywords:** atypical bartonellosis, *Bartonella henselae*, bartonellosis, cat scratch disease, hepatosplenic abscesses

## Abstract

In most cases, infection due to *Bartonella henselae* causes a mild disease presenting with a regional lymphadenopathy frequently associated with a low-grade fever, headache, poor appetite and exhaustion that spontaneously resolves itself in a few weeks. As the infection is generally transmitted by cats through scratching or biting, the disease is named cat scratch disease (CSD). However, in 5–20% of cases, mainly in immunocompromised patients, systemic involvement can occur and CSD may result in major illness. This report describes a case of systemic CSD diagnosed in an immunocompetent 4-year-old child that can be used as an example of the problems that pediatricians must solve to reach a diagnosis of atypical CSD. Despite the child’s lack of history suggesting any contact with cats and the absence of regional lymphadenopathy, the presence of a high fever, deterioration of their general condition, increased inflammatory biomarkers, hepatosplenic lesions (i.e., multiple abscesses), pericardial effusion with mild mitral valve regurgitation and a mild dilatation of the proximal and medial portion of the right coronary artery, seroconversion for *B. henselae* (IgG 1:256) supported the diagnosis of atypical CSD. Administration of oral azithromycin was initiated (10 mg/kg/die for 3 days) with a progressive normalization of clinical, laboratory and US hepatosplenic and cardiac findings. This case shows that the diagnosis of atypical CSD is challenging. The nonspecific, composite and variable clinical features of this disease require a careful evaluation in order to achieve a precise diagnosis and to avoid both a delayed diagnosis and therapy with a risk of negative evolution.

## 1. Background

In most cases, infection due to *Bartonella henselae* causes a mild disease presenting with a regional lymphadenopathy frequently associated with a low-grade fever, headache, poor appetite and exhaustion that spontaneously resolves itself in a few weeks [1]. As infection is generally transmitted by cats through scratching or biting [2], the disease is named cat scratch disease (CSD). However, in 5–20% of cases, mainly in immunocompromised patients, systemic involvement can occur and CSD may result in major illness [3]. A prolonged high fever can develop and be associated with a number of severe clinical manifestations. Skin, eyes, liver, spleen, heart and nervous and musculoskeletal systems can be involved. In these cases, prompt diagnosis and proper treatment are mandatory to avoid immediate and serious long-term risks for the patient [3]. Unfortunately, due to the variability of signs and symptoms and the lack of gold standard criteria, a diagnosis of systemic CSD can be both difficult and significantly delayed. Atypical CSD can evolve completely uncontrolled for several days [4]. For a rapid diagnosis, a high index of suspicion is needed: clinical features and compatible epidemiological context should be accurately evaluated, the most reliable laboratory tests should be performed and results must be rationally evaluated.

This report describes a case of systemic CSD diagnosed in an immunocompetent child that can be used as an example of the problems that pediatricians must solve to reach a diagnosis of atypical CSD.

## 2. Case Presentation

A 4-year-old boy with unremarkable medical history was admitted to the hospital due to a 5-day intermittent fever peaking up to 41 °C and associated with abdominal pain. In the period preceding hospitalization, the child had received only paracetamol when their body temperature was above 38.5 °C. Moreover, although in close contact with pets, family members did not recall the child having been scratched or bitten. No bites by fleas, keds, lice, sand flies, ticks, mites or spiders were reported. 

At admission, the physical examination revealed an axillary temperature of 39.9 °C, a blood pressure of 90/60 mm Hg, a heart rate of 96 beats per minute and a respiratory rate of 18 breaths per minute. A slight bilateral cervical lymphadenopathy was noted. No skin lesion ascribable to cat scratches or bites was detectable on any exposed surface. The chest and abdomen examination did not result in any pathological finding. The liver edge was palpable at the right costal margin. The spleen was not palpable. Laboratory tests showed a normal and complete blood count (Hb 12.8 g/dL, white blood cells 9350/mm^3^, neutrophils 46.5%, platelets 217,000/mm^3^), but there was a significant increase in serum acute phase reactants values (C-reactive protein (CRP) 56.6 mg/L, procalcitonin (PCT) 13.84 ng/mL). Serum aspartate aminotransferase and alanine aminotransferase showed an increase (101 U/L and 107 U/L, respectively), whereas the results of all the other laboratory tests including proteinogram, complement fraction, coagulation markers and α-fetoprotein were within the normal range (Table 1). Eye examination was normal. Chest X-ray did not show any abnormalities, but the lung ultrasound (US) revealed a slight bilateral pleural effusion; echocardiography was normal. The abdomen ultrasound revealed slight hepatosplenomegaly, enlarged hilum-hepatic lymph nodes, acalculous cholecystitis and the presence of pericholecystic fluid.

Extensive laboratory tests to exclude infections and lymphoproliferative disorders were performed. In particular, a Mantoux test and interferon-gamma release assay (IGRA) for tuberculosis and serologic testing for *B. henselae*, cytomegalovirus, *Toxoplasma gondii*, *Mycoplasma pneumoniae*, Epstein–Barr virus, adenovirus, HCV, enterovirus, *Shigella*, *Yersinia*, *Salmonella typhi*, *Brucella* and *Coxiella burnetii* infections were performed. A blood sample for bacterial culture was collected. While awaiting results, an empiric intravenous antibiotic therapy with piperacillin–tazobactam (150 mg/kg/die in three doses) was started. 

**Figure 1:**
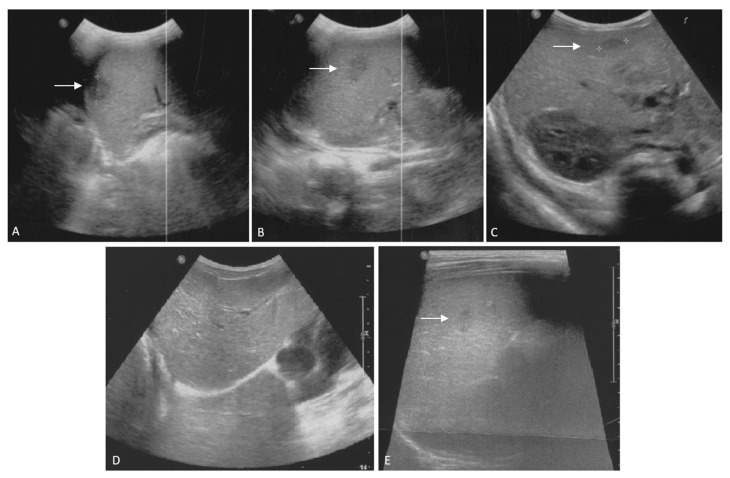
Liver and spleen ultrasound scans of the patient on admission (**A**–**C**) and during follow-up (**D**,**E**) in a 4-year-old immunocompetent child with atypical bartonellosis. Admission. (**A**,**B**): enlarged inhomogeneous spleen with multiple hypoechoic lesions (**A**): ∅ 15 mm; (**B**): ∅ 17 mm). (**C**): liver appears of increased echogenicity, with multiple hypoechoic lesions; the arrow indicates the larger one (∅ 15 mm) in hepatic segment V. Enlargement of hilar hepatic lymphnodes can be appreciated. After one month from admission. (**D**,**E**): abdominal ultrasound shows the disappearance of the hepatic lesions (**D**) and the persistence of two hypoechoic spleen lesions, in decline (**E**): largest lesion, ∅ 7 mm, previously ∅ 17 mm as shown in (**B**).

On the third day of hospitalization, with all laboratory tests specifically planned to identify infectious agents receiving negative results, persistence of a fever up to 39 °C in the early morning and afternoon and a slight but substantial deterioration of the child’s general conditions, we repeated both blood tests and the US. Results showed mild hypoalbuminemia and hyponatremia, with a persistent high level of CRP and PCT, and an elevated erythrocyte sedimentation rate (Table 1). The abdominal US showed the onset of multiple hypoechoic liver and spleen lesions with a target appearance and irregular margins (Figure 1), suggesting multiple hepatosplenic abscesses. Moreover, the presence of fluid in abdominal recesses was evidenced. A second echocardiography showed the appearance of pericardial effusion, mild mitral valve regurgitation and a mild dilatation of the proximal and medial portion of the right coronary artery (respectively, Z-score +2.53 and +2.31), with a normal value of the myocardial contractility (75%). Piperacillin–tazobactam was discontinued and a combined antibiotic therapy with intravenous meropenem (100 mg/kg/die in three doses) and intravenous vancomycin (40 mg/kg/die in three doses) was started. Screening for *B. henselae* was repeated, this time with positive results (IgG 1:256). 

Despite the lack of history suggesting contact with cats, due to the presence of hepatosplenic lesions (i.e., multiple abscesses) in the absence of any different potential etiology, the diagnosis of atypical CSD was considered highly likely and administration of oral azithromycin was initiated (10 mg/kg/die for 3 days). Clinical, laboratory and US hepatosplenic and cardiac findings progressively normalized. The repetition of the *B. henselae* antibody after one month confirmed the titer detected during the hospitalization (IgG 1:256) and the last abdominal US revealed the disappearance of the hypoechoic liver lesions and the persistence of two hypoechoic spleen lesions, which were in decline.

## 3. Discussion

The case reported here highlights how difficult the diagnosis of atypical CSD can be and how only a high index of suspicion can allow the prescription of an effective therapy in a short time period. Understanding the complex interactions between *B. henselae*, its vectors and its reservoirs, as well as the breadth of infection by *B. henselae* around the world, will help to assess the impact of bartonellosis on public health [5]. In addition to transmission through domestic animals, numerous different vectors transmit Bartonella species. These vectors include fleas, keds, lice, sand flies and potentially ticks, mites and spiders [5]. Atypical CSD is relatively uncommon in younger children and this can be, per se, a limit to suspect the disease, particularly, as in this case, when no significant lymphadenopathy is present. In the USA, among the 224 cases described between 2005 and 2014, only 36.2% were diagnosed in children <14 years of age [3]. Moreover, gold standard criteria for a definitive diagnosis have not been established. An attempt was made some years ago by Margileth [6], who suggested that a definitive diagnosis could be made if, in a patient, the following four criteria could be satisfied: (1) cat or flea contact with or without a scratch mark or a regional inoculation lesion; (2) negative tests for other potential causes of adenopathy or infection, including polymerase chain reaction (PCR) tests on blood or tissue samples and computed tomography scans of liver and spleen; (3) a positive enzyme immunoassay (EIA) or indirect fluorescent antibody assay serology test > 1:64 for *B. henselae;* (4) a biopsy of a lymph node, skin, liver, bone or eye showing granulomatous inflammation compatible with CSD. However, considering the patient history, reliability of laboratory tests and difficulties in performing biopsies, it seems highly unlikely that all these criteria can be simultaneously satisfied in a patient. Even if contact with a cat is common, skin lesions due to scratches or bites are detected in only 60% of documented CSD cases [7]. Moreover, poor attention is generally paid to the potential role of dogs in *B. henselae* infection transmission as these pets have been found implicated in approximately 5% of cases [8]. Serologic tests, such as both EIAs and indirect immunofluorescence assays, have suboptimal sensitivity and specificity and can lead to incorrect results in a relevant number of cases [9]. The low specificity is ascribed to the high seroprevalence in the normal population due to cross reactivity with *Coxiella burnetii* infection, *Chlamydophila infection* and non-*henselae Bartonella* infections [10,11]. The low sensitivity appears to be strictly associated with the distribution of different *B. henselae* genotypes. Serologic tests are more effective in the detection of antibodies against genotype 1, and, if genotype 2 is the cause of disease, tests can be negative despite a true infection [9]. Moreover, a prior infection can cause false positive results. Limitations can also derive from the use of PCR tests to detect *B. henselae* in blood or in tissue biopsies. Despite the fact that reliable results of DNA amplification methods for the identification of *B. henselae* have been reported [12], in the same cases of atypical CSD recently described, PCR tests were repeatedly found unable to detect the pathogen [13]. Finally, the authorization to perform biopsies for histological assessments may be delayed or even denied by the parents of children with a supposed infection. 

In the case described here, all the criteria proposed by Margileth could not be satisfied [6]. Diagnosis was suspected starting from the splenic and liver US findings, the evidence that most of the infectious diseases with clinical manifestation resembling CSD could be excluded and the lack of response to broad-spectrum antibiotic therapy. Hepatosplenic involvement occurs in around one quarter of atypical CSD cases with children <14 years of age at increased risk (relative risk (RR) 1.76, 95% confidence interval (CI) 1.04–2.99) [6]. When present, micro-abscesses are considered as evidence of atypical CSD, particularly when patients have persistent fever, abdominal pain and characteristic abdominal US findings, as in the patient here reported. The evidence of a positive EIA test was later considered a further element for diagnosis as it was the temporal association between the administration of azithromycin and the prompt resolution of clinical manifestations. 

In this case, together with hepatosplenic lesions, significant cardiac involvement was found. This is relatively uncommon and once again highlights the difficulties for a proper diagnosis of atypical CSD. In the aforementioned USA study, only 3.6% of cases presented with cardiac signs and symptoms [3]. Endocarditis is the most common cardiac diagnosis. It is described more frequently in adults, though a previous valvular disease can favor this complication in children [14]. However, other heart manifestations have been associated as part of the clinical picture of atypical CSD. Myocarditis and pericarditis have been described [15]. Moreover, as *B. henselae* has been found able to infect endothelial cells and reduce their number and functionality, it has been supposed that this pathogen could play a role in the determination of acute and long-term vascular alterations [16]. 

Finally, in this child, a strict temporal relationship between the use of azithromycin and disease resolution was found. The real importance of the prescribed antibiotic treatment for the resolution of this case could be debated, as the efficacy of antibiotics in atypical CSD cases is not precisely defined and official recommendations are lacking. Suggestions for antibiotic treatment are usually based only on personal experience and expert opinion [16]. However, the rapid improvement of all clinical manifestations of the disease, including the most severe ones, such as hepatic and splenic micro-abscesses, seems to indicate a possible direct effect of the antibiotic therapy. Regarding antibiotic use, despite the fact that these drugs are not recommended in mild to moderate typical CSD cases, their administration is suggested in atypical cases, particularly when they occur in immunocompromised subjects. A group of drugs including macrolides, rifampin, ciprofloxacin, trimethoprim–sulfamethoxazole and gentamicin are considered of choice [17,18,19,20]. Other antibiotics, including those initially prescribed in the reported patient, are not recommended as, despite being effective in vitro, they were not effective in vivo in typical CSD cases [21]. Unfortunately, the dosages and duration of treatment are not established. In addition, new light was shed on the treatment of bartonellosis owing to a recent publication that showed a non-coding RNA that controlled the transcription of a gene encoding a DNA-binding protein that modulates biofilm development in *B. henselae* [22].

## 4. Conclusions

This case shows that the diagnosis of atypical CSD is challenging. The nonspecific, composite and variable clinical features of this disease require a careful evaluation in order to achieve a precise diagnosis and avoid both a delayed diagnosis and a risk of negative evolution. In the case of a prolonged fever of unknown origin, when abdominal pain, hepatosplenic lesions on US and heart abnormalities are present, a *B. henselae* infection must be considered even if a suggestive history or a clear lymphadenopathy is lacking. In these cases, antibiotic treatment with drugs considered effective against *B. henselae* must be prescribed.

## Figures and Tables

**Table 1:** Laboratory findings on admission and during follow-up in a 4-year-old immunocompetent child with atypical bartonellosis.

Variable	On Admission	Time from Admission						15 Days after Discharge
		Day 3	Day 5	Day 10	Day 16	Day 20	Day 24	
Hemoglobin (g/dL)	12.8	10.5	10	11.1	11.5	11.0	11.7	11.8
Hematocrit (%)	37.6	32	30	34.3	35.2	34	36.5	35.6
White cell count (per μL)	9350	7770	9860	8920	5140	6330	6870	6370
Neutrophils (%)	46.5	42.3	59.3	25.7	26	21.2	18.6	21.6
Limphocytes (%)	44.1	47.7	29.5	56.6	53.5	59.6	68.3	66.1
Monocytes (%)	3.8	9.5	10.8	14.3	11.8	15	8.4	9.3
Eosinophils (%)	0.1	0.0	0.1	3.0	3.1	3.3	4.1	2.7
Basophils (%)	0.7	0.5	0.3	0.4	1.3	0.9	0.6	0.3
Red-cell count (per μL)	4.75 × 10^6^	3.98 × 10^6^	3.78 × 10^6^	4.26 × 10^6^	4.37 × 10^6^	4.27 × 10^6^	4.65 × 10^6^	4.62 × 10^6^
Platelet count (per μL)	217,000	200,000	328,000	587,000	540,000	483,000	521,000	398,000
Sodium (mEq/L)	131	134	-	135	133	132	136	137
Potassium (mEq/L)	4	4.2	-	4.6	5.1	5.1	4.8	4.3
Chloride (mEq/L)	99	106	-	102	101	100	102	103
Calcium (mg/dL)	9.0	8.0	-	9.0	9.2	9.6	10.2	9.8
Urea nitrogen (mg/dL)	29	12	-	26	41	41	30	38
Creatinine (mg/dL)	0.5	0.3	-	0.4	0.3	0.3	0.2	0.4
Glucose (mg/dL)	105	103	-	120	75	86	80	84
Albumin (g/dL)	-	3.0	-	3.3		3.7	3.9	4.8
ALT (U/L)	101	70	-	36	28	47	39	23
AST (U/L)	107	62	-	21	50	64	58	45
CRP (mg/L)	57.6	47.5	65.9	-	11.2	-	1.2	0.5
PCT (ng/mL)	13.84	10.38	3.64	-	-	-	0.06	0.02
ESR (mm/hr)	-	95	-	-	86	-	-	36

ALT, alanine aminotransferase; AST, aspartate aminotransferase; ESR, erythrocyte sedimentation rate; CRP, C-reactive protein; PCT, procalcitonin.

## Data Availability

All the available data were reported in the Case presentation.
